# Symptomatic Management of Febrile Illnesses in Children: A Systematic Review and Meta-Analysis of Parents' Knowledge and Behaviors and Their Evolution Over Time

**DOI:** 10.3389/fped.2018.00279

**Published:** 2018-10-05

**Authors:** Nathalie Bertille, Edward Purssell, Nils Hjelm, Natalya Bilenko, Elena Chiappini, Eefje G. P. M. de Bont, Michael S. Kramer, Philippe Lepage, Sebastiano A. G. Lava, Santiago Mintegi, Janice E. Sullivan, Anne Walsh, Jérémie F. Cohen, Martin Chalumeau

**Affiliations:** ^1^Inserm U1153 Équipe de Recherche en Épidémiologie Obstétricale, Périnatale et Pédiatrique, Centre de Recherche Épidémiologie et Statistique Sorbonne Paris Cité, Université Paris Descartes, Paris, France; ^2^Department of General Pediatrics and Pediatric Infectious Diseases, Hôpital Necker-Enfants malades, AP-HP, Université Paris Descartes, Paris, France; ^3^School of Health Sciences, City, University of London, London, United Kingdom; ^4^Department of Public Health, Faculty of Health Sciences, Ben-Gurion University of the Negev, Beersheba, Israel; ^5^Department of Health Science, University of Florence, Anna Meyer Children's University Hospital, Florence, Italy; ^6^Department of Family Medicine, CAPHRI School for Public Health and Primary Care, Maastricht University, Maastricht, Netherlands; ^7^Department of Epidemiology, Biostatistics and Occupational Health, McGill University, Montreal, QC, Canada; ^8^Department of Pediatrics, McGill University, Montreal, QC, Canada; ^9^Hôpital Universitaire des Enfants Reine Fabiola, Université Libre de Bruxelles, Bruxelles, Belgium; ^10^University Children's Hospital Bern-University of Bern, Bern, Switzerland; ^11^Division of Clinical Pharmacology and Toxicology, Institute of Pharmacological Sciences of Southern Switzerland, Ente Ospedaliero Cantonale, Lugano, Switzerland; ^12^Pediatric Emergency Department, Cruces University Hospital, Bilbao, Spain; ^13^Biocruces Health Research Institute, University of the Basque Country, Bilbao, Spain; ^14^Department of Pediatrics, University of Louisville, Louisville, KY, United States; ^15^Institute of Health and Biomedical Innovation, Queensland University of Technology, Brisbane, QLD, Australia

**Keywords:** health behavior, child, fever, parents, meta-analysis

## Abstract

Recommendations to guide parents' symptomatic management of febrile illnesses in children have been published in many countries. The lack of systematic appraisal of parents' knowledge and behaviors and their evolution over time precludes an analysis of their impact and identification of targets for future educational messages. We systematically searched for studies published between 1980 and 2016 that reported a quantitative evaluation of knowledge and behaviors of >50 parents for managing fever in children. We used MEDLINE and tracked related articles, citations and co-authors personal files. Study selection and data extraction were independently performed by two reviewers. For each item of knowledge and behaviors, we calculated mean frequencies during the first and last quinquennials of the studied period and assessed temporal trends with inverse-variance weighted linear regression of frequencies over years. We observed substantial methodological heterogeneity among the 62 included articles (64 primary studies, 36,791 participants, 30 countries) that met inclusion criteria. Statistically significant changes over time were found in the use of rectal (98 to 4%) and axillary temperature measurement (1–19%), encouraging fluid intake (19–62%), and use of acetylsalicylic acid (60 to 1%). No statistically significant change was observed for the accurate definition of fever (38–55%), or the use of acetaminophen (91–92%) or ibuprofen (20–43%). Parents' knowledge and behaviors have changed over time but continue to show poor concordance with recommendations. Our study identified future targets for educational messages, including basic ones such as the definition of fever.

## Introduction

Fever, one of the most common symptoms of illness in children, is frequently caused by self-limited viral infections. Despite not being harmful in itself, fever may be associated with pain and discomfort (1, 2). However, fever is often the first cause for medical consultation in childhood, and the main source of drug exposure in pediatrics (1–12). Although widely used and potentially helpful to relieve discomfort, antipyretic medications are not without possible side-effects. Several studies have highlighted that parents' knowledge and behaviors are largely dominated by “fever phobia,” the undue anxiety about fever, more than 40 years after it was first described (1, 5–16). Notably, parents are often scared by the rare situations in which fever is the first sign of a serious illness, such as severe bacterial infection (17), or is associated with seizures (1, 5).

Recommendations to guide parents' symptomatic management of febrile illnesses in children have been published by several national health agencies and medical societies in a number of different countries (2, 18–24). Generally, recommendations address (Table 1): (i) temperature measurement method [rectally (18, 19, 23, 24), orally (18, 19, 23, 24), auricular (2, 18, 19, 21–23), or axillary (2, 18, 19, 21–24)]; (ii) the definition of fever [temperature ≥38°C (2, 3, 18, 19, 23, 24)]; (iii) when to start antipyretics (2, 18, 19, 21–24); (iv) physical treatments [encouraging fluid intake (2, 18, 19, 22–24), light clothing 2, 18, 19, 22–24, adjust room temperature (19, 23, 24)]; and (v) drug treatments [monotherapy with acetaminophen (2, 3, 18, 19, 21–24) or ibuprofen (2, 3, 18, 19, 22–24)]. The lack of systematic appraisal of temporal changes in parents' knowledge and behaviors was recently highlighted as an obstacle to evaluate the impact of recommendations (25). Such a systematic appraisal could help identify the knowledge and behaviors that differ from recommendations in order to develop future educational messages (25).

**Table 1:** Examples of recommendations for the symptomatic management of febrile illnesses in children.

	**Canada (18)**	**France (19)**	**Italy (20, 21)**	**UK (22)**	**USA (23)**	**WHO (24)**
**TEMPERATURE MEASUREMENT METHOD**						
Rectal	Yes	Yes	No	No	Yes	Yes
Oral	Yes	Yes	No	No	Yes	Yes
Auricular	Yes	Yes	Yes	Yes	Yes	No
Axillary	Yes	Yes	Yes	Yes	Yes	Yes
**FEVER DEFINITION**						
≥38°C	Yes	Yes	^*^	Yes	Yes	Yes
**WHEN TO START DRUG TREATMENT**						
Based on a fever threshold	No	Yes	No	No		Yes
Based on symptoms	Yes	Yes	Yes	Yes	Yes	Yes
**PHYSICAL TREATMENTS**						
Adjust room temperature		Yes	No	No	Yes	Yes
Light clothing	Yes	Yes	No	Yes	Yes	Yes
Encourage fluid intake	Yes	Yes	No	Yes	Yes	Yes
Others			No		Sponging	
**DRUG TREATMENT**						
Monotherapy as first-line treatment	Yes	Yes	Yes	Yes		Yes
Acetaminophen	Yes	Yes	Yes	Yes	Yes	Yes
Ibuprofen	Yes	Yes	Yes	Yes	Yes	Yes
Acetylsalicylic acid	No	Yes	No	No	No	No

* *Empty cases are not clearly addressed in recommendations*.

We aimed to systematically review studies on parents' knowledge and behaviors for the management of febrile illnesses in children, and to assess whether they have changed over time.

## Methods

We conducted a systematic review following the methodology proposed by the Centre for Reviews and Dissemination (26) and reported it using the Preferred Reporting Items for Systematic Reviews and Meta-Analyses (PRISMA) guideline (27) (checklist in Appendix 1 in Supplementary Material).

### Search strategy and selection criteria

We aimed to include all studies performed since 1980 [date of the first publication on fever phobia (6)] that reported quantitative data on knowledge and behaviors regarding fever in children (aged < 18 years) of >50 (arbitrary) parents. We searched for study reports published from January 1, 1980 to September 1, 2016, in MEDLINE via PubMed, with the following search strategy: (“behavior” [MeSH Term] OR “attitude” [MeSH Term] OR “analgesics” [MeSH Term] OR “acetaminophen/therapeutic use” [Mesh Term]) AND (“child” [MeSH Term] OR “infant” [MeSH Term]) AND “fever” [MeSH Term] AND (“1980/01/01” [PDAT]: “2016/09/01” [PDAT]). We also searched Science Citation Index and Google Scholar for studies citing the included studies and examined the first 50 “related articles” of included studies in PubMed. Finally, we hand-searched the reference lists of included studies, as well as personal files of the international network of co-authors. We included studies published in English, French, German, Italian, and Spanish.

Two review authors (NB, NH) independently excluded clearly ineligible studies based on their titles and abstracts. They then retrieved the full text of potentially eligible articles to independently evaluate them for inclusion. Another review author (MC) acted as arbiter in case of discrepancies between them.

### Data extraction

For each included study, two review authors (NB, NH) independently extracted study characteristics (year of publication and of the study, general methodology of the survey, country, sample size) and the frequency reported for the following knowledge and behaviors, which were selected based on their citation in published recommendations (Table 1): temperature measurement method (rectally, orally, auricular, or axillary), definition of fever, physical treatments (encouraging fluid intake, light clothing, adjust room temperature, bathing, or sponging) and drug treatments (monotherapy, use of acetaminophen, ibuprofen, or acetylsalicylic acid).

### Risk of bias assessment

For observational studies, no tool has been validated to evaluate the risks of bias and threat to generalizability for conducting a systematic review. After discussions among the authors, the following criteria were selected to assess these risks: single- vs. multi- center recruitment, hospital-based vs. broader recruitment of participants, and theoretical knowledge and behaviors (case scenarios) vs. observed ones for a current/recent case. Multicenter not only hospital-based studies evaluating observed knowledge and behaviors for current/recent cases were considered as being at low risk of bias and threat to generalizability. Studies with one or more of the following characteristics were considered at high risk of bias and threat to generalizability: having a single center sample, being only hospital-based, or based on case scenarios.

### Statistical analyses

To describe “current” parents' knowledge and behaviors, we performed a fixed-effect meta-analysis by calculating an inverse-variance weighted mean frequency from studies performed during the last decade. Then, we tested for temporal changes in parents' knowledge and behaviors using inverse-variance weighted linear regression of frequency over time (in years). To describe those temporal changes, we reported mean frequencies of the first and last quinquennials over the study periods based on available data (the study periods may change across the different knowledge and behavior domains assessed).

Analyses were first performed using all included studies. Subsequently, we carried out a sensitivity analysis by restriction to studies with low risk of bias. For the sensitivity analysis, the number of studies was insufficient for calculating a mean frequency during the last decade and for analyzing temporal changes. Then, we compared parents' knowledge and behaviors during the last decade according to the economic development status of the country where the study was performed: countries with advanced economies (CAE) vs. countries with emerging and developing economies (CEE) (28). Lastly, we stratified the analyses of temporal trends according to the economic development status. All analyses were carried out using Stata v11 (StataCorp, College Station, Texas, USA).

## Results

### Study selection and characteristics

**Figure 1:**
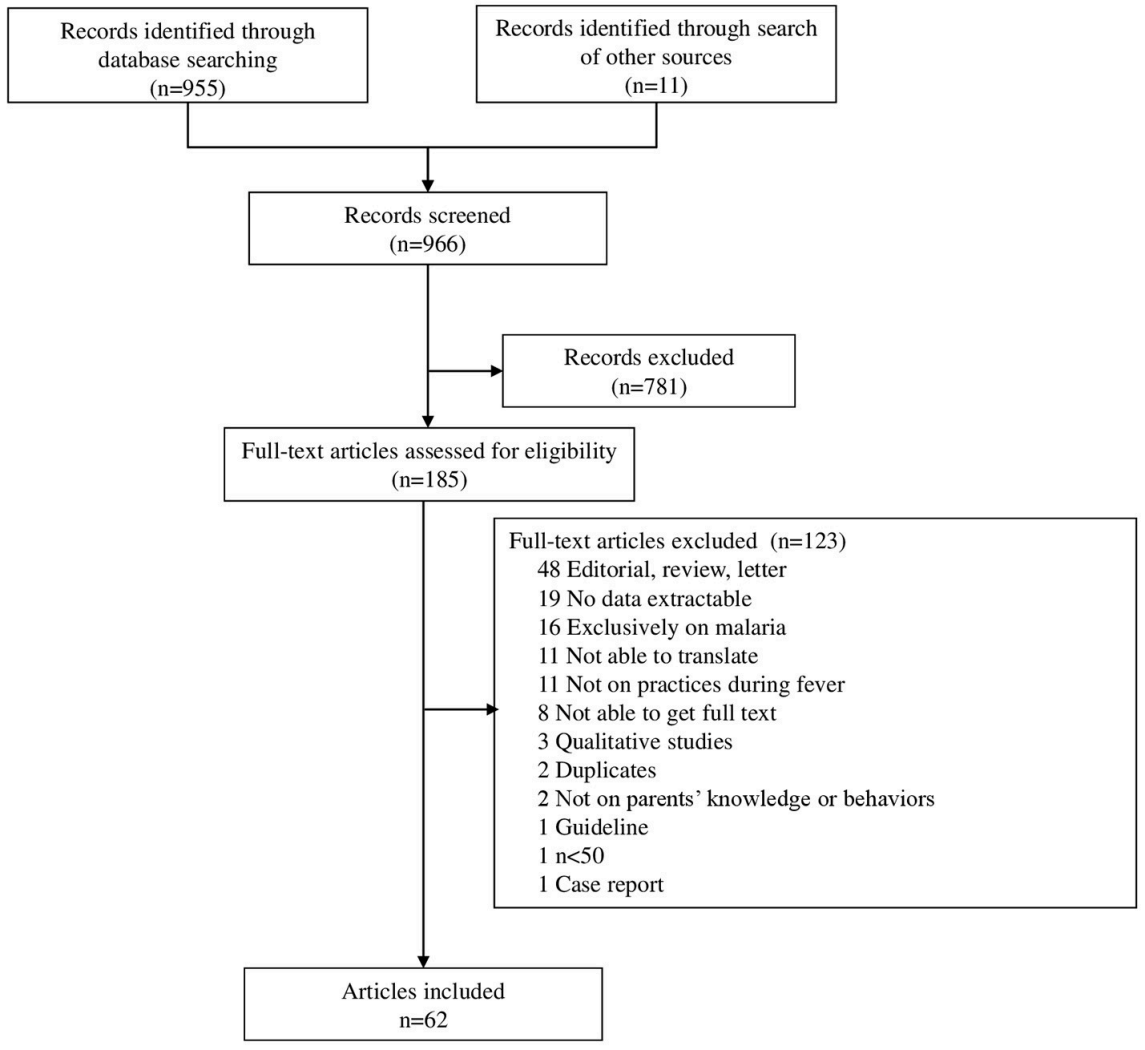
Study flow diagram.

The search identified 966 articles (Figure 1) that were screened for eligibility on titles and abstracts (*n* = 781) and full texts (*n* = 185). We identified 62 articles that met our inclusion criteria (Appendix 2 in Supplementary Material), corresponding to 64 primary studies published since 1980, reporting the knowledge and behaviors of 36,791 parents in 30 countries, including 47 studies (73%; *n* = 2 6,138) performed in CAE, of which 14 (22%; *n* = 2,705) were from the USA (Appendixes 3–5 in Supplementary Material). The main parental attitudes and behaviors studied were temperature measurement methods (37/64; 58%), definition of fever (27/64; 42%), physical treatments (44/64; 69%), and drug treatments (41/64; 64%). The weighted mean age of pediatric participants, reported in 11/62 articles, was 22 months.

### Risk of bias of included studies

Thirty-one (48%) studies had single-center recruitment, 38 (59%) were hospital-based only, and 44 (69%) were based on theoretical knowledge and behaviors (case scenarios) (Appendixes 4, 5 in Supplementary Material). Five studies (8%; *n* = 12,334) were judged to be at low risk of bias (12, 29–32).

### Parents' knowledge and behaviors

The weighted means of temperature measurement methods reported in studies conducted in the last decade were (Table 2): touching the child 42%, (95% confidence interval –CI–: 29–55%), and use of axillary 33% (95% CI: 12–54%), auricular 6% (95% CI: 1–11%), rectal 5% (95% CI: 0–14%), and oral 1% (95% CI: 0–2%) thermometer. A statistically significant reduction over time was observed in the use of rectal measurement method (from 98% in the first quinquennial period to 4% in the last one; *p* < 0.01), with a corresponding increased use of axillary measurement (from 1 to 19%, *p* < 0.01).

**Table 2:** Parents' knowledge and behaviors concerning febrile illnesses in children.

	**Countries with advanced economies**				**Countries with emerging and developing economies**				**All countries**			
**(%)**	**Last decade^a^**	**1stquinquennial^b^**	**Last quinquennial^b^**	**P^c^**	**Last decade^a^**	**1st quinquennial^b^**	**Last quinquennial^b^**	**P^c^**	**Last decade^a^**	**1st quinquennial^b^**	**Last quinquennial^b^**	**P^c^**
**TEMPERATURE MEASUREMENT METHOD**												
Rectal	57	98	53	< 0.01	1	1	1	0.79	5	98	4	< 0.01
Oral	2	1	2	0.39	0	0	0	0.35	1	1	1	0.96
Auricular	18	8	14	0.35	2	2	2	0.59	6	9	6	0.88
Axillar	8	1	10	0.17	85	91	85	0.10	33	1	19	< 0.01
Touching	38	17	38	0.04	44	52	44	0.13	42	17	42	0.08
**FEVER DEFINITION**												
Temperature ≥38°C	60	46	60	0.37	54	30	54	0.32	58	38	55	0.27
**PHYSICAL TREATMENTS**												
Encourage fluid intake	72	19	34	0.03	79	79^d^	79^d^	0.92	73	19	62	0.01
Light clothing	64	19	63	0.03	35	21	35	0.61	48	19	47	0.17
Adjust room temperature	20	16	20	0.97	NA	NA	NA	NA	20	16	20	0.97
Bathe	31	32	28	0.46	69	36	69	0.02	36	32	34	0.25
Sponge	24	50	64	0.70	66	80	62	0.53	36	38	67	0.62
**DRUG TREATMENTS**												
Monotherapy	65	53	71	0.46	75	65	75	0.87	67	53	71	0.22
Acetaminophen	92	91	98	< 0.01	71	83	71	0.03	87	91	92	0.09
Ibuprofen	32	20	41	0.87	23	13	24	0.09	28	20	43	0.72
AAS	1	60	1	0.07	2	4	2	0.09	1	60	1	0.02

a Inverse-variance-weighted pooled frequency in the last decade;

b Inverse-variance-weighted pooled frequency of the first and last quinquennials with available data;

c P-value of linear regression of inverse-variance-weighted frequencies over time (in years);

d *Same period of study; NA, No data available; AAS, acetylsalicylic acid*.

**Figure 2:**
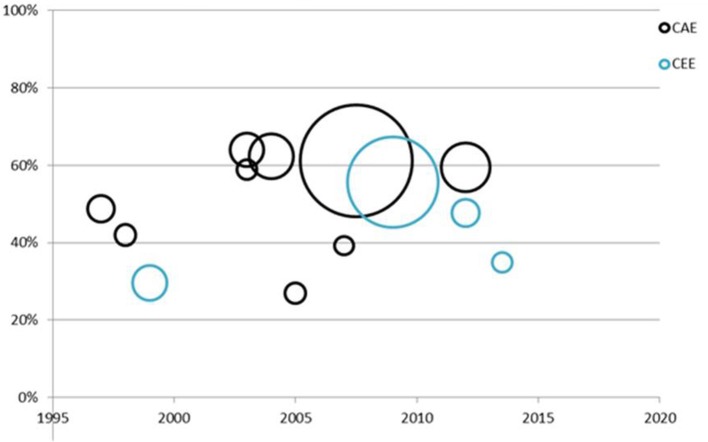
Time trend of the frequency of parents citing 38°C as the threshold for the definition of fever in children. CAE, countries with advanced economies; CEE, countries with emerging and developing economies. Each point represents one study; dot size is proportional to inverse of the variance and thus the study weight in the regression.

The threshold commonly recommended for defining fever (i.e., 38°C) was known currently by 58% (95% CI: 52–64%) of parents (Figure 2, Table 2). We observed a statistically non-significant increase over time in the frequency of a definition of fever concordant with recommendations, from 38 to 55% (*p* = 0.27).

Common physical treatments used by parents were (Table 2): encouraging fluid intake (73%, 95% CI: 56–90%), light clothing (48%, 95% CI: 27–69%), sponging the child (36%, 95% CI: 19–53%), bathing (36%, 95% CI: 15–56%), and adjust room temperature (20%, 95% CI: 0–100%). A significant increase in encouraging fluid intake was observed over time (from 19 to 62%, *p* = 0.01).

Drug monotherapy was used by 67% (95% IC: 55–79%) of parents in studies conducted in the last decade (Table 2). The drugs used were: acetaminophen (87%, 95% CI: 78–96%), ibuprofen (28%, 95% CI: 15–40%), and acetylsalicylic acid (1%, 95% CI: 0–2%). A significant decrease was observed over time in the use of acetylsalicylic acid (from 60 to 1%, *p* = 0.02). No statistically significant changes were observed for acetaminophen (91 to 92%, *p* = 0.09) and ibuprofen (20 to 43%, *p* = 0.72).

### Sensitivity analysis for studies with low risk of bias

Only one study with low risk of bias was conducted during the last decade (*n* = 6,596) (12). The temperature measurement methods were axillary (6%), auricular (19%), rectal (64%), and oral (2%) thermometer. The recommended threshold used for defining fever was known by 61% of parents. Physical treatments used by parents were: encouraging fluid intake (78%), light clothing (62%), adjust room temperature (27%), bathing (20%), and sponging the child (17%). A drug monotherapy was used by 66% of parents: acetaminophen (78%), ibuprofen (29%), and acetylsalicylic acid (2%).

### Stratified analyses according to countries' economic development

In CAE, we observed more frequent use of rectal measurement (57 vs. 1% in CAE and CEE, respectively, *p* < 0.001), oral measurement (2 vs. 0%, *p* = 0.02), auricular measurement (18 vs. 2%, *p* < 0.001), light clothing (64 vs. 35%, *p* = 0.004), and acetaminophen (92 vs. 71%, *p* < 0.001), but less frequent use of axillary measurement (8 vs. 85%, *p* < 0.001), sponging (24 vs. 66%, *p* < 0.001), and bathing (31 vs. 69%, *p* = 0.001).

In CAE, a significant increase over time was observed in the frequency of touching the child to measure fever (from 17 to 38%, *p* = 0.04), encouraging fluid intake (from 19 to 34%, *p* = 0.03), light clothing (from 19 to 63%, *p* = 0.03), and use of acetaminophen (from 91 to 98%, *p* < 0.01), along with a significant reduction in use of the rectal measurement (from 98 to 53%, *p* < 0.01). In contrast, studies performed in CEE showed a significant increase in bathing (from 36 to 69%, *p* = 0.02) and a significant reduction in use of acetaminophen (from 83 to 71%, *p* = 0.03).

## Discussion

### Summary of evidence

Published studies show an important and persistent gap between parents' knowledge and behaviors for the symptomatic management of febrile illnesses in children, on the one hand, and recommendations by health agencies and medical societies, on the other (2, 18, 23). “Fever phobia” seems to persist, with very frequent (>80%) use of antipyretic drugs, including in studies with low risk of bias and threat to generalizability, and both in countries with advanced and less advanced economies. The high frequency of antipyretic use could be justified if it aimed at making the child more comfortable, however most studies did not identify the goal of drug use, and in the few that reported it, 10–60% of parents reported using drugs to control the level of temperature rather than relieve discomfort (11, 15, 33–35). Frequent discordance between parents' knowledge and behaviors and recommendations were also observed for the measurement of temperature by touch (42% in studies performed during the last decade), a definition of fever different from 38°C (58%), and the use of bathing or sponging (36%). The data on temperature measurement by touch should be interpreted with caution, however, because the phrasing of the questions often did not allow distinguishing the exclusive use of touch and its use before confirmation by recommended methods. Important gains over time in the frequency of concordance with recommendations were reported for encouraging fluid intake (from 19–62%) and use of acetylsalicylic acid (from 60 to 1%).

We also observed differences between countries with advanced or less advanced economies. In CEE, we observed more frequent use of axillary temperature measurement (85% compared to 8% in CAE, respectively), bathing (69 vs. 31%), and sponging (66 vs. 24%) but less frequent use of drugs (acetaminophen: 71 vs. 92%; and ibuprofen 23 vs. 32%). These differences may be related to economic and cultural barriers and/or limited accessibility to current recommendations. Given the lack of detailed information on cultural background of population analyzed in included studies and ongoing recommendations in the region where they were performed, we were not able to formulate precise hypothesis that could explain these differences. However, it is well-known that the rectal route is not traditionally used for temperature measurement and drug administration in some countries, for cultural reason.

### Implications for clinical practice and public health

Our study shows that effort is still required to de-dramatize fever among parents. Several reasons may explain the persisting gap between recommendations and parents' knowledge and behaviors, including insufficient evidence for recommendations and consequent discrepancies among them on key aspects of fever management, the low readability of patient educational tools, poor dissemination of guidelines (2), incorrect or suboptimal counseling and examples provided by healthcare providers (36), and resistance to change (such as positive experiences with non-recommended practices based on older children). Health education interventions concerning fever in children may need to use alternative methods (such as mobile apps for smartphones and tablets, printed papers, advises during medical appointments, television spots, or e-campaigns) to reach parents and need to provide simple and clear guidance, especially for parents with a low educational level. The results of our systematic review and meta-analysis suggest that these renewed educational efforts should include knowledge as basic as the definition of fever (which was known by only 58% of parents −61% in CAE, 54% in CEE- in the last decade). The lack of international consensus on the threshold for defining fever is a barrier to the standardization of educational programs.

### Implications for research

One of the potential barriers to parents' uptake of educational messages is the lack of international consensus on some aspects of symptomatic management of febrile illnesses, including methods for measuring body temperature, the definition of child discomfort and clear indications for physical and drug treatments (2, 3, 18, 19, 21, 23, 24). For example (Table 1), some health agencies or scientific societies do not mention or discourage rectal temperature measurement, or the use of ibuprofen or rectal acetaminophen, while others still encourage them (2, 3, 20, 21, 24). This lack of consensus may reflect cultural differences in practices such as the use of the rectal route, genuine uncertainty in the case of acetaminophen or ibuprofen, or the fact that many recommendations appear pragmatic rather than evidence-based. A consensus on such areas would be helpful in reducing parental anxiety, which may actually be increased by differences in guidelines. As guidance is increasingly focused on the use of drugs to reduce discomfort, research on the dimensions of discomfort in the feverish child and tools to measure them would help operationalize these recommendations.

Only five studies (< 10%) were at low risk of bias and only one of those was published in the last 10 years (12). As fever is a main source of drug consumption and consultation in children, future studies should try to minimize risk of bias and threats to generalizability by improving their designs.

### Limitations

We arbitrarily excluded studies with < 50 participants and those published before 1980. However, only one study was excluded because of insufficient number of participants (Figure 1), so this arbitrary choice probably had only a marginal influence on our results. We searched for publications only in MEDLINE, Google Scholar, and Science Citation Index and only those written in English, French, German, Italian, or Spanish. The strength and the direction of the bias related to these identification process and inclusion criteria are unclear.

Only five studies (< 10%) had a low risk of bias (multicenter not only hospital-based studies evaluating observed attitudes for current/recent cases), only one of which dates from the last 10 years. Thus our findings may not be valid for current parents.

The use of linear regression to explore temporal changes in parents' knowledge and behaviors is arguable, as it may lack statistical power and tests only for linear trends. However, more classical meta-regression would have provided even lower statistical power to detect changes over time, given the limited number of available studies.

We were unable to restrict our analyses to younger children. Such a restriction may well modify parents' behaviors, owing to differences in practical issues (e.g., type of temperature measurement methods) or because fever phobia is more frequent among parents of very young children (37).

## Conclusion

Despite significant changes over time, parents' knowledge and behaviors showed poor concordance with recommendations for the symptomatic management of febrile illnesses in their children. Our study identified main targets for future educational messages including basic ones such as definition of fever. This is likely to become more important over time as there is a general trend toward encouraging self-management of common conditions in infants and children by their families. Any educational message should avoid giving false reassurance about fever, as in a small number of cases it is an early symptom of more severe disease. Studies with low risk of bias that adequately evaluate current parents' knowledge and attitudes behaviors and an international consensus on basic recommendations are needed.

## Author contributions

NBe conceptualized and designed the study, carried out the study selection, the data extraction, and the analyses, and drafted the manuscript. NH carried out the study selection, and the data extraction, and critically reviewed the manuscript. EC and EP conceptualized, designed the study, and critically reviewed the manuscript. NBi, EdB, AW, JS, MK, PL, SL, and SM critically reviewed the study protocol, participated in study identification, and critically reviewed the manuscript. JC critically reviewed the study protocol, statistical analyses, and drafted the manuscript. MC conceptualized and designed the study, and drafted the manuscript. All authors approved the final manuscript as submitted.

### Conflict of interest statement

The authors declare that the research was conducted in the absence of any commercial or financial relationships that could be construed as a potential conflict of interest. The handling Editor declared a past co-authorship with one of the authors SM.
