# Supplementary material for: Symptomatic Management of Febrile Illnesses in Children: A Systematic Review and Meta-Analysis of Parents' Knowledge and Behaviors and Their Evolution Over Time
Source: Front Pediatr. 2018 Oct 5;6:279. doi: 10.3389/fped.2018.00279 (PMC6183237; doi:10.3389/fped.2018.00279)
Supplement: Supplementary file 2 [file Data_Sheet_2.doc]

**Appendix 2: References of included articles (alphabetic order)**

Al-Eissa YA, Al-Sanie AM, Al-Alola SA, Al-Shaalan MA, Ghazal SS, Al-Harbi AH, et al. Parental perceptions of fever in children. Ann Saudi Med 2000;20:202-5.

Al-Nouri L, Basheer K. Mothers' perceptions of fever in children. J Trop Pediatr 2006;52:113-6.

Ames JT, Hayden GF, Campbell RE, Lohr JA. Parents' conception of their use of over-the-counter medicines. Clin Pediatr (Phila) 1982;21:298-301.

Arica SG, Arica V, Onur H, Gulbayzar S, Dag H, Obut O. Knowledge, attitude and response of mothers about fever in their children. Emerg Med J 2012;29:e4.

Asekun-Olarinmoye EO, Egbewale BE, Olajide FO. Subjective assessment of childhood fever by mothers utilizing primary health care facilities in Osogbo, Osun State, Nigeria. Niger J Clin Pract 2009;12:434-8.

Athamneh L, El-Mughrabi M, Athamneh M, Essien EJ, Abughosh S. Parents' knowledge, attitudes and beliefs of childhood fever management in Jordan: A cross-sectional study. J Appl Res Child 2014;5.

Belin J, Lablache Combier B, Reinert P. [Parent-pediatrician synergism faced with childhood fever]. Ann Pediatr (Paris) 1992;39:588-92.

Bertille N, Fournier-Charriere E, Pons G, Chalumeau M. Managing fever in children: a national survey of parents' knowledge and practices in France. PLoS ONE 2013;8:e83469.

Betz MG, Grunfeld AF. 'Fever phobia' in the emergency department: a survey of children's caregivers. Eur J Emerg Med 2006;13:129-33.

Bilenko N, Tessler H, Okbe R, Press J, Gorodischer R. Determinants of antipyretic misuse in children up to 5 years of age: a cross-sectional study. Clin Ther 2006;28:783-93.

Blumenthal I. What parents think of fever. Fam Pract 1998;15:513-8.

Boivin JM, Weber F, Fay R, Monin P. [Management of paediatric fever: is parents' skill appropriate?]. Arch Pediatr 2007;14:322-9.

Calvo Monge C, Onis Gonzalez E, Vazquez Ronco M, Martinez Gonzalez M, Benito Fernandez J, Mintegui Raso S. [Current use of antipyretics in children at home]. An Esp Pediatr 2001;54:607-8.

Chang MC, Chen YC, Chang SC, Smith GD. Knowledge of using acetaminophen syrup and comprehension of written medication instruction among caregivers with febrile children. J Clin Nurs 2012;21:42-51.

Chiappini E, Parretti A, Becherucci P, Pierattelli M, Bonsignori F, Galli L, et al. Parental and medical knowledge and management of fever in Italian pre-school children. BMC Pediatr 2012;12:97.

Cinar ND, Altun I, Altinkaynak S, Walsh A. Turkish parents' management of childhood fever: a cross-sectional survey using the PFMS-TR. Australas Emerg Nurs J 2014;17:3-10.

Cohee LM, Crocetti MT, Serwint JR, Sabath B, Kapoor S. Ethnic differences in parental perceptions and management of childhood fever. Clin Pediatr (Phila) 2010;49:221-7.

Crocetti M, Moghbeli N, Serwint J. Fever phobia revisited: have parental misconceptions about fever changed in 20 years? Pediatrics 2001;107:1241-6.

Crocetti M, Sabath B, Cranmer L, Gubser S, Dooley D. Knowledge and management of fever among Latino parents. Clin Pediatr (Phila) 2009;48:183-9.

de Bont EG, Francis NA, Dinant GJ, Cals JW. Parents' knowledge, attitudes, and practice in childhood fever: an internet-based survey. Br J Gen Pract 2014;64:e10-6.

Dong L, Jin J, Lu Y, Jiang L, Shan X. Fever phobia: a comparison survey between caregivers in the inpatient ward and caregivers at the outpatient department in a children's hospital in China. BMC Pediatr 2015;15:163.

Emmerton L, Chaw XY, Kelly F, Kairuz T, Marriott J, Wheeler A, et al. Management of children's fever by parents and caregivers: Practical measurement of functional health literacy. J Child Health Care 2013.

Enarson MC, Ali S, Vandermeer B, Wright RB, Klassen TP, Spiers JA. Beliefs and expectations of Canadian parents who bring febrile children for medical care. Pediatrics 2012;130:e905-12.

Erkek N, Senel S, Sahin M, Ozgur O, Karacan C. Parents' perspectives to childhood fever: comparison of culturally diverse populations. J Paediatr Child Health 2010;46:583-7.

Grass L, Claudet I, Oustrk S, Bros B. Knowledge and behaviour of parents during an episode of fever [in French]. Rev. Prat. Med. Gen 2005;686-87:381-4.

Gribetz B, Cronley SA. Underdosing of acetaminophen by parents. Pediatrics 1987;80:630-3.

Impicciatore P, Nannini S, Pandolfini C, Bonati M. Mother's knowledge of, attitudes toward, and management of fever in preschool children in Italy. Prev Med 1998;27:268-73.

Jackowska T, Sapała-Smoczyńska A, Rurarz A, Nowicka K. Parents’ knowledge of fever and management procedures in the case of its occurrence in children under 12 years of age. Postępy Nauk Medycznych 2014.

Jensen JF, Tonnesen LL, Soderstrom M, Thorsen H, Siersma V. Paracetamol for feverish children: parental motives and experiences. Scand J Prim Health Care 2010;28:115-20.

Karwowska A, Nijssen-Jordan C, Johnson D, Davies HD. Parental and health care provider understanding of childhood fever: a Canadian perspective. CJEM 2002;4:394-400.

Kelly L, Morin K, Young D. Improving caretakers' knowledge of fever management in preschool children: Is it possible? J Pediatr Health Care 1996;10:167-73.

Kilmon CA. Parents' knowledge and practices related to fever management. J Pediatr Health Care 1987;1:173-9.

Kramer MS, Naimark L, Leduc DG. Parental fever phobia and its correlates. Pediatrics 1985;75:1110-3.

Kwak YH, Kim do K, Jang HY, Kim JJ, Ryu JM, Oh SB, et al. Fever phobia in Korean caregivers and its clinical implications. J Korean Med Sci 2013;28:1639-44.

Langer T, Pfeifer M, Sonmez A, Tarhan B, Ostermann T. Appraisal of childhood fever by German and Turkish mothers in Germany--results of a pilot study. Turk J Pediatr 2010;52:471-80.

Linder N, Sirota L, Snapir A, Eisen I, Davidovitch N, Kaplan G, et al. Parental knowledge of the treatment of fever in children. Isr Med Assoc J 1999;1:158-60.

Matziou V, Brokalaki H, Kyritsi H, Perdikaris P, Gymnopoulou E, Merkouris A. What Greek mothers know about evaluation and treatment of fever in children: an interview study. Int J Nurs Stud 2008;45:829-36.

McErlean MA, Bartfield JM, Kennedy DA, Gilman EA, Stram RL, Raccio-Robak N. Home antipyretic use in children brought to the emergency department. Pediatr Emerg Care 2001;17:249-51.

Nijman RG, Oostenbrink R, Dons EM, Bouwhuis CB, Moll HA. Parental fever attitude and management: influence of parental ethnicity and child's age. Pediatr Emerg Care 2010;26:339-42.

Pereira GL, Tavares NU, Mengue SS, Pizzol Tda S. Therapeutic procedures and use of alternating antipyretic drugs for fever management in children. J Pediatr (Rio J) 2013;89:25-32.

Poirier MP, Collins EP, McGuire E. Fever phobia: a survey of caregivers of children seen in a pediatric emergency department. Clin Pediatr (Phila) 2010;49:530-4.

Polat M, Kara S, Tezer H, Tapisiz A, Derinoz O, Dolgun A. A current analysis of caregivers' approaches to fever and antipyretic usage. J Infect Dev Ctries 2014;8:365-71.

Purssell E. Parental fever phobia and its evolutionary correlates. J Clin Nurs 2009;18:210 - 8.

Raffaeli G, Orenti A, Gambino M, Peves Rios W, Bosis S, Bianchini S, et al. Fever and Pain Management in Childhood: Healthcare Providers' and Parents' Adherence to Current Recommendations. Int J Environ Res Public Health 2016;13.

Rkain M, Rkain I, Safi M, Kabiri M, Ahid S, Benjelloun BD. Knowledge and management of fever among Moroccan parents. East Mediterr Health J 2014;20:397-402.

Rodríguez Serna A, Astobiza Beobide E, González Balenciaga M, Azkunaga Santibáñez B, Benito Fernández J, Mintegi Raso S. [Change in habits among the population in the management of childhood fever]. An Pediatr (Barc) 2006;64:497-8.

Rupe A, Ahlers-Schmidt CR, Wittler R. A comparison of perceptions of fever and fever phobia by ethnicity. Clin Pediatr (Phila) 2010;49:172-6.

Saettini F, Bettinelli A. Fever phobia among Italian caregivers: a survey in a pediatric emergency department. Minerva Pediatr 2014;66:261-6.

Sakai R, Niijima S, Marui E. Parental knowledge and perceptions of fever in children and fever management practices: differences between parents of children with and without a history of febrile seizures. Pediatr Emerg Care 2009;25:231-37.

Sakai R, Okumura A, Marui E, Niijima S, Shimizu T. Does fever phobia cross borders? The case of Japan. Pediatr Int 2012;54:39-44.

Sellier-Joliot C, Di Patrizio P, Minary L, Boivin JM. [AFSSAPS 2005 recommendations have not modified the way parents take care of children's fever]. Arch Pediatr 2015;22:352-9.

Singhi S, Padmini P, Sood V. Urban parents' understanding of fever in children: its dangers, and treatment practices. Indian Pediatr 1991;28:501-5.

Soltani M, Sallem I, Elmhamdi S, Bouslah M, Sriha A, Ben Salem K. Connaissances et pratiques des mères de la région de Monastir (Tunisie) devant la fièvre de l'enfant. Rev. Tun. Infec 2009:19--28.

Stagnara J, Vermont J, Durr F, Ferradji K, Mege L, Duquesne A, et al. [Parents' attitudes towards childhood fever. A cross-sectional survey in the Lyon metropolitan area (202 cases)]. Presse Med 2005;34:1129-36.

Taveras EM, Durousseau S, Flores G. Parents' beliefs and practices regarding childhood fever: a study of a multiethnic and socioeconomically diverse sample of parents. Pediatr Emerg Care 2004;20:579-87.

Teagle AR, Powell CV. Is fever phobia driving inappropriate use of antipyretics? Arch Dis Child 2014;99:701-2.

Tran TKL. Fever management in children: Vietnamese parents' and paediatric nurses' knowledge, beliefs and practices [Philosophy]: Queensland University of Technology; 2014.

Wallenstein MB, Schroeder AR, Hole MK, Ryan C, Fijalkowski N, Alvarez E, et al. Fever literacy and fever phobia. Clin Pediatr (Phila) 2013;52:254-9.

Walsh A, Edwards H, Fraser J. Over-the-counter medication use for childhood fever: a cross-sectional study of Australian parents. J Paediatr Child Health 2007;43:601-6

Walsh A, Edwards H, Fraser J. Parents' childhood fever management: community survey and instrument development. J Adv Nurs 2008;63:376-88.

Wright AD, Liebelt EL. Alternating antipyretics for fever reduction in children: an unfounded practice passed down to parents from pediatricians. Clin Pediatr (Phila) 2007;46:146-50.

Zyoud SH, Al-Jabi SW, Sweileh WM, Nabulsi MM, Tubaila MF, Awang R, et al. Beliefs and practices regarding childhood fever among parents: a cross-sectional study from Palestine. BMC Pediatr 2013;13:66.
