# Supplementary material for: Symptomatic Management of Febrile Illnesses in Children: A Systematic Review and Meta-Analysis of Parents' Knowledge and Behaviors and Their Evolution Over Time
Source: Front Pediatr. 2018 Oct 5;6:279. doi: 10.3389/fped.2018.00279 (PMC6183237; doi:10.3389/fped.2018.00279)
Supplement: Supplementary file 3 [file Data_Sheet_3.doc]

Appendix 3: Countries where included studies were performed, according to their

economic development status

| **Economic development status of the country** | **Countries** | **Number of study** |
| --- | --- | --- |
| **Countries with emerging and developing economies** | Brazil | 1 |
|  | China | 1 |
|  | India | 1 |
|  | Iraq | 1 |
|  | Jordan | 1 |
|  | Morocco | 1 |
|  | Nigeria | 1 |
|  | Palestine | 1 |
|  | Poland | 1 |
|  | Saudi-Arabia | 1 |
|  | Tunisia | 1 |
|  | Turkey | 4 |
|  | United Arab Emirates | 1 |
|  | Vietnam | 1 |
|  | **Sub-total** | 17 |
| **Countries with advanced economies.** | Australia | 3 |
|  | Canada | 3 |
|  | Denmark | 1 |
|  | France | 6 |
|  | Germany | 1 |
|  | Greece | 1 |
|  | Israel | 2 |
|  | Italy | 4 |
|  | Japan | 2 |
|  | Korea | 1 |
|  | Netherlands | 2 |
|  | New Zealand | 1* |
|  | Spain | 3 |
|  | Taiwan | 1 |
|  | United Kingdom | 3 |
|  | United States of America | 14 |
|  | **Sub-total** | 47 |

* One study reported results from surveys performed in Australia and New-Zealand

(Emmerton L, et al. 2013)
