# Supplementary material for: Symptomatic Management of Febrile Illnesses in Children: A Systematic Review and Meta-Analysis of Parents' Knowledge and Behaviors and Their Evolution Over Time
Source: Front Pediatr. 2018 Oct 5;6:279. doi: 10.3389/fped.2018.00279 (PMC6183237; doi:10.3389/fped.2018.00279)
Supplement: Supplementary file 4 [file Data_Sheet_4.doc]

Appendix 4: Parents’ knowledge and behaviors for the symptomatic management of febrile illnesses in children in countries with emerging and developing economies.

| **Author, date** | **Country** | **N** |  | **Temperature measurement method** | | | | | | | |  | | **Fever definition** |  | **Physical treatments** | | | | | | | | |  | | | | | | **Drug treatments** | | | | | | | |
| --- | --- | --- | --- | --- | --- | --- | --- | --- | --- | --- | --- | --- | --- | --- | --- | --- | --- | --- | --- | --- | --- | --- | --- | --- | --- | --- | --- | --- | --- | --- | --- | --- | --- | --- | --- | --- | --- | --- |
|  |  |  |  | **Rectal** | **Oral** | **Ax** | | **Aur** | | **Touch** | |  | | **T=38°C** |  | **Fluid** | | **Clothe** | | **Room** | | **Bathe** | | **Sponge** | | |  | | | **Mono** | | | | **Ace** | | **Ibu** | | **AAS** |
| *Single center studies* | | | | | | | | | | | | | | | | | | | | | | | | | | | | | | | | | | | | | | |
| **Singhi, 1991*** | India | 100 |  |  |  |  | |  | |  | |  | |  |  |  |  | |  | |  | | 29 | |  | | | |  | | | |  | |  | |  | |
| **Al Nouri, 2006*** | Iraq | 100 |  |  |  |  | |  | | 92 | |  | |  |  |  | 5 | |  | | 27 | | 98 | |  | | | |  | | | | 94 | |  | | 22 | |
| **Betz, 2006*^§^** | United Arab Emirates | 264 |  | 7 | 19 | 60 | | 14 | |  | |  | |  |  |  |  | |  | | 22 | | 93 | |  | | | | 87 | | | | 92 | | 10 | |  | |
| **Erkek, 2010*^§^** | Turkey | 200 |  |  |  |  | |  | | 57 | |  | |  |  |  | 76 | |  | |  | | 79 | |  | | | |  | | | | 86 | | 12 | |  | |
| **Arica, 2012*^§^** | Turkey | 4500 |  | 1 | 0 | 91 | | 2 | | 49 | |  | | 56 |  |  | 20 | |  | |  | |  | |  | | | |  | | | | 82 | | 14 | | 4 | |
| **Athamneh, 2014** | Jordan | 419 |  | 37 | 16 | 43 | | 9 | | 36 | |  | | 48 |  |  |  | |  | |  | | 76 | |  | | | |  | | | | 65 | | 16 | | 4 | |
| **Jackowska, 2014*** | Poland | 200 |  |  |  |  | |  | |  | |  | | 35 |  |  |  | |  | |  | | 67 | |  | | | | 48 | | | | 62 | | 65 | | 12 | |
| **Rkain, 2014*** | Morocco | 264 |  | 74 |  | 25 | | 1 | | 44 | |  | |  |  | 16 | 21 | |  | | 47 | | 16 | |  | | | |  | | | | 86 | | 5 | | 9 | |
| **Dong, 2015*** | China | 621 |  |  |  |  | |  | |  | |  | |  |  |  |  | |  | |  | | 83 | |  | | | | 93 | | | | 14 | | 84 | | 0 | |
| *Multicenter studies* | | | | | | | | | | | | | | | | | | | | | | | | | | | | | | | | | | | | | | |
| **Al-Eissa, 2000*^§^** | Saudi-Arabia | 560 |  |  |  | |  |  | |  | |  | | 30 |  |  |  | |  | |  | |  | |  | | | |  | | | |  | |  | |  | |
| **Asekun-Olarinmoye, 2009** | Nigeria | 300 |  | 0 | 15 | | 80 |  | |  | |  | |  |  |  |  | |  | | 57 | | 19 | |  | | | |  | | | |  | |  | |  | |
| **Soltani, 2009** | Tunisia | 491 |  | 76 |  | |  | |  | |  | |  |  |  |  |  | |  | |  | | 83 | | |  | |  | | | |  | | |  | |  | |
| **Zyoud, 2013** | Palestine | 402 |  | 26 | 50 | | 21 | |  | | 65 | |  |  |  |  |  | |  | |  | | 50 | | |  | |  | | | |  | | |  | |  | |
| **Pereira, 2013** | Brazil | 630 |  |  | 0 | | 93 | |  |  | |  | |  |  |  |  | |  | |  | |  | |  | | | 45 | | | | |  | |  | |  | |
| **Cinar, 2014^§^** | Turkey | 205 |  |  |  | |  | |  |  | |  | |  |  | 67 |  | |  | |  | |  | |  | | | |  | | | |  | |  | |  | |
| **Polat, 2014*^§^** | Turkey | 1032 |  | 0 | 8 | | 57 | | 19 | | 16 | |  |  |  |  | 12 | |  | | 76 | |  | | |  | | 59 | | | | 60 | | | 68 | | 4 | |
| **Tran, 2014*^§^** | Vietnam | 365 |  |  |  | | 91 | |  | | 82 | |  |  |  | 95 | 96 | |  | |  | | 81 | | |  | | 59 | | | |  | | |  | |  | |

Otherwise stated data are percentages; *hospital recruitment only; ^§^current case; Rectal: rectally; Oral: oral; Ax: axillary; Aur: auricular; Touch: touching; T: temperature; Fluid: encourage fluid intake; Clothe: light clothing; Room: adjust room temperature; Bathe: bathing; Sponge: sponging; Mono: monotherapy; Ace: acetaminophen; Ibu: ibuprofen; AAS: acetylsalicylic acid
