# Supplementary material for: Symptomatic Management of Febrile Illnesses in Children: A Systematic Review and Meta-Analysis of Parents' Knowledge and Behaviors and Their Evolution Over Time
Source: Front Pediatr. 2018 Oct 5;6:279. doi: 10.3389/fped.2018.00279 (PMC6183237; doi:10.3389/fped.2018.00279)
Supplement: Supplementary file 5 [file Data_Sheet_5.doc]

**Appendix 5: Parents’ knowledge and behaviors for the symptomatic management of febrile illnesses in children in co**untries with advanced economies.

| **Author, date** | **Country** | **N** |  | **Temperature measurement method** | | | | | |  | **Fever definition** |  | **Physical treatments** | | | | | | | |  | **Drug treatments** | | | | | | |
| --- | --- | --- | --- | --- | --- | --- | --- | --- | --- | --- | --- | --- | --- | --- | --- | --- | --- | --- | --- | --- | --- | --- | --- | --- | --- | --- | --- | --- |
|  |  |  |  | **Rectal** | **Oral** | **Ax** | **Aur** | **Touch** | |  | **T=38°C** |  | **Fluid** | | **Clothe** | **Room** | | | **Bathe** | **Sponge** |  | **Mono** | | **Ace** | | **Ibu** | | **AAS** |
| *Single center studies* | | | | | | | | | | | | | | | | | | | | | | | | | | | | |
| **Ames, 1982*** | U.S.A. | 109 |  |  |  |  |  | |  |  |  |  |  |  | | |  |  | |  |  |  | 49 | |  | |  | |
| **Kramer, 1985^§^** | Canada | 202 |  |  |  |  |  | |  |  |  |  |  |  | | |  |  | |  |  |  |  | |  | |  | |
| **Gribetz, 1987*^§^** | U.S.A. | 96 |  |  |  |  |  | |  |  |  |  |  |  | | |  | 16 | |  |  |  | 97 | |  | | 3 | |
| **Kilmon, 1987** | U.S.A. | 100 |  |  |  |  |  | |  |  |  |  |  |  | | |  |  | | 50 |  |  | 87 | |  | | 8 | |
| **Blumenthal, 1998*** | U.K. | 392 |  | 8 | 8 | 84 |  | |  |  |  |  |  |  | | |  |  | | 70 |  |  |  | |  | |  | |
| **Calvo Monge, 2001*** | Spain | 200 |  |  |  | 84 | 5 | |  |  |  |  |  |  | | |  |  | |  |  | 50 | 56 | |  | |  | |
| **McErlean, 2001*^§^** | U.S.A. | 138 |  |  |  |  |  | |  |  |  |  |  |  | | |  |  | |  |  |  |  | |  | | 0 | |
| **Bilenko, 2006*^§^** | Israel | 201 |  |  |  |  |  | |  |  |  |  |  |  | | |  |  | |  |  | 91 | 97 | | 9 | |  | |
| **Rodriguez Serna, 2006*** | Spain | 200 |  | 10 |  | 84 |  | |  |  |  |  |  |  | | |  |  | |  |  |  | 56 | | 18 | | 20 | |
| **Rodriguez Serna, 2006*** | Spain | 421 |  | 13 |  | 77 |  | |  |  |  |  |  |  | | |  |  | |  |  |  | 45 | | 49 | | 1 | |
| **Wright, 2007*** | U.S.A. | 256 |  |  |  |  |  | |  |  |  |  |  |  | | |  |  | |  |  | 52 | 70 | | 64 | |  | |
| **Matziou, 2008*^§^** | Greece | 327 |  |  |  | 95 |  | |  |  |  |  | 86 |  | | |  | 33 | | 65 |  |  |  | |  | |  | |
| **Crocetti, 2009*** | U.S.A. | 180 |  | 16 | 15 | 68 | 1 | |  |  |  |  |  |  | | |  |  | | 73 |  | 96 |  | |  | |  | |
| **Purssell, 2009*** | U.K. | 181 |  |  |  |  |  | | 25 |  |  |  | 75 |  | | |  |  | | 34 |  | 61 | 93 | | 51 | |  | |
| **Chang, 2010*^§^** | Taiwan | 102 |  |  |  | 49 | 45 | |  |  |  |  |  |  | | |  |  | |  |  |  |  | |  | |  | |
| **Nijman, 2010*^§^** | Netherlands | 211 |  | 83 |  |  |  | |  |  |  |  |  |  | | |  |  | |  |  |  |  | |  | |  | |
| **Poirier, 2010*** | U.S.A. | 230 |  | 50 | 60 | 47 | 17 | | 7 |  |  |  |  |  | | |  |  | |  |  |  |  | |  | |  | |
| **Rupe, 2010*** | U.S.A. | 348 |  | 13 | 35 | 72 | 11 | | 23 |  |  |  |  | 46 | | |  | 47 | |  |  |  | 76 | | 44 | | 0 | |

Otherwise stated data are percentages; *hospital recruitment only; §current case; Rectal: rectally; Oral: oral; Ax: axillary; Aur: auricular; Touch: touching; T: temperature; Fluid: encourage fluid intake; Clothe: light clothing; Room: adjust room temperature; Bathe: bathing; Sponge: sponging; Mono: monotherapy; Ace: acetaminophen; Ibu: ibuprofen; AAS: acetylsalicylic acid.

**Appendix 5: Parents’ knowledge and behaviors^1^ for the symptomatic management of febrile illnesses in children in countries with advanced economies** (continued).

| **Author, date** | **Country** | **N** | |  | **Temperature measurement method** | | | | | |  | **Fever definition** |  | | **Physical treatments** | | | | | | |  | **Drug treatments** | | | | |
| --- | --- | --- | --- | --- | --- | --- | --- | --- | --- | --- | --- | --- | --- | --- | --- | --- | --- | --- | --- | --- | --- | --- | --- | --- | --- | --- | --- |
|  |  |  | |  | **Rectal** | **Oral** | **Ax** | **Aur** | **Touche** | |  | **T=38°C** | |  | | **Fluid** | **Clothe** | | **Room** | **Bathe** | **Sponge** |  | **Mono** | **Ace** | | **Ibu** | **AAS** |
| **Sakai, 2012*** | Japan | 211 | |  |  |  |  |  | |  |  |  | |  | | 30 |  | |  |  |  |  |  |  | |  |  |
| **Saettini, 2014*** | Italy | 98 | |  |  |  |  |  | |  |  |  | |  | |  |  | |  |  |  |  |  | 93 | | 6 |  |
| **Teagle, 2014*** | U.K. | 78 | |  |  |  |  |  | |  |  |  | |  | | 87 |  | |  |  | 64 |  |  |  | |  |  |
| **Raffaeli, 2016*** | Italy | 464 | |  | 21 |  | 66 | 6 | |  |  |  | |  | |  |  | |  |  |  |  | 87 | 88 | |  |  |
| *Multicenter studies* | | | | | | | | | | | | | | | | | | | | | | | | | | | |
| **Belin, 1992^§^** | France | 4624 |  | | 98 | 1 | 1 |  |  | |  |  | |  | | 49 | 50 | 16 | | 32 |  |  | 53 | 64 |  | | 71 |
| **Kelly, 1996** | U.S.A. | 86 |  | |  |  |  |  |  | |  |  | |  | |  |  |  | |  |  |  |  | 98 |  | | 1 |
| **Impicciatore, 1998^§^** | Italy | 707 |  | |  |  | 65 |  |  | |  |  | |  | | 2 | 2 |  | |  | 10 |  |  | 32 |  | | 1 |
| **Linder, 1999** | Israel | 650 |  | | 51 | 16 | 14 |  |  | |  |  | |  | |  |  |  | | 2 |  |  |  | 96 |  | |  |
| **Crocetti, 2001*** | U.S.A. | 340 |  | |  |  |  |  |  | |  |  | |  | |  |  |  | |  |  |  |  |  |  | |  |
| **Karwowska, 2002** | Canada | 209 |  | |  |  |  |  |  | |  |  | |  | |  | 5 |  | |  |  |  |  | 85 | 10 | | 0 |
| **Taveras, 2004** | U.S.A. | 235 |  | |  |  |  |  |  | |  | 42 | |  | |  | 59 |  | |  |  |  |  | 93 | 59 | | 7 |
| **Grass, 2005** | France | 556 |  | |  |  |  |  |  | |  | 64 | |  | | 86 | 89 | 41 | | 75 |  |  |  | 64 |  | |  |
| **Stagnara, 2005^§^** | France | 202 |  | | 82 |  | 4 | 9 |  | |  | 59 | |  | | 24 | 41 |  | | 65 |  |  |  | 93 | 39 | |  |
| **Boivin, 2007** | France | 1038 |  | | 91 | 6 | 9 |  | 42 | |  | 62 | |  | | 30 | 65 | 7 | | 94 |  |  | 21 | 92 | 64 | | 16 |
| **Walsh, 2007** | Australia | 401 |  | |  |  |  |  |  | |  |  | |  | |  |  |  | |  |  |  | 44 | 94 | 7 | |  |
| **Walsh, 2008** | Australia | 401 |  | |  |  |  |  |  | |  |  | |  | | 49 | 44 |  | |  | 38 |  |  |  |  | |  |
| **Sakai, 2009*** | Japan | 386 |  | |  |  |  |  |  | |  |  | |  | | 88 |  |  | |  |  |  |  |  |  | |  |
| **Cohee, 2010*** | U.S.A. | 307 |  | | 10 | 60 | 20 | 14 | 4 | |  |  | |  | |  |  |  | |  | 72 |  |  |  |  | |  |
| **Cohee, 2010*** | U.S.A. | 180 |  | | 8 | 25 | 38 | 4 | 28 | |  |  | |  | |  |  |  | |  | 73 |  |  |  |  | |  |

Otherwise stated data are percentages; *hospital recruitment only; §current case; Rectal: rectally; Oral: oral; Ax: axillary; Aur: auricular; Touch: touching; T: temperature; Fluid: encourage fluid intake; Clothe: light clothing; Room: adjust room temperature; Bathe: bathing; Sponge: sponging; Mono: monotherapy; Ace: acetaminophen; Ibu: ibuprofen; AAS: acetylsalicylic acid.

**Appendix 5: Parents’ knowledge and behaviors^1^ for the symptomatic management of febrile illnesses in children in countries with advanced economies** (continued).

| **Author, date** | **Country** | **N** |  | **Temperature measurement method** | | | | |  | **Fever definition** |  | **Physical treatments** | | | | | |  | **Drug treatments** | | | | |
| --- | --- | --- | --- | --- | --- | --- | --- | --- | --- | --- | --- | --- | --- | --- | --- | --- | --- | --- | --- | --- | --- | --- | --- |
|  |  |  |  | **Rectal** | **Oral** | **Ax** | **Aur** | **Touch** |  | **T=38°C** |  | **Fluid** | **Clothe** | **Room** | | **Bathe** | **Sponge** |  | **Mono** | | **Ace** | **Ibu** | **AAS** |
| **Jensen, 2010** | Denmark | 100 |  |  |  |  |  |  |  |  |  |  |  | |  |  |  |  |  | 61 | |  |  |
| **Langer, 2010*^§^** | Germany | 50 |  | 72 | 4 | 17 | 28 |  |  |  |  |  | 80 | |  | 14 | 78 |  |  |  | |  |  |
| **Chiappini, 2012** | Italy | 388 |  | 16 | 1 | 82 | 7 |  |  |  |  |  |  | |  |  |  |  | 83 | 97 | | 29 | 1 |
| **Enarson, 2012** | Canada | 776 |  |  |  |  |  |  |  |  |  |  | 73 | |  | 65 | 80 |  |  |  | |  |  |
| **Bertille, 2013^§^** | France | 6596 |  | 64 | 2 | 6 | 19 |  |  | 61 |  | 78 | 62 | | 27 | 20 | 17 |  | 67 | 78 | | 29 | 2 |
| **Kwak, 2013*^§^** | Korea | 746 |  |  |  |  | 58 |  |  |  |  |  |  | |  |  |  |  | 64 |  | |  |  |
| **Wallenstein, 2013*** | U.S.A. | 100 |  |  |  |  |  |  |  | 0 |  |  |  | |  |  |  |  |  |  | |  |  |
| **De Bont, 2014** | Netherlands | 625 |  | 77 | 1 | 1 | 17 | 64 |  |  |  |  |  | |  |  |  |  |  |  | |  |  |
| **Emmerton, 2014** | Australia,  New Zealand | 417 |  |  |  |  |  |  |  |  |  |  |  | |  |  |  |  |  | 84 | | 16 |  |
| **Sellier-Joliot, 2015** | France | 1273 |  | 71 | 5 | 20 | 29 | 52 |  | 60 |  | 28 | 72 | | 7 | 74 |  |  | 31 | 99 | | 74 | 1 |

Otherwise stated data are percentages; *hospital recruitment only; §current case; Rectal: rectally; Oral: oral; Ax: axillary; Aur: auricular; Touch: touching; T: temperature; Fluid: encourage fluid intake; Clothe: light clothing; Room: adjust room temperature; Bathe: bathing; Sponge: sponging; Mono: monotherapy; Ace: acetaminophen; Ibu: ibuprofen; AAS: acetylsalicylic acid.
